# Racial difference in Acylation Stimulating Protein (ASP) correlates to triglyceride in non-obese and obese African American and Caucasian women

**DOI:** 10.1186/1743-7075-6-18

**Published:** 2009-04-17

**Authors:** Thea Scantlebury-Manning, Joseph Bower, Katherine Cianflone, Hisham Barakat

**Affiliations:** 1 Department of Biological and Chemical Sciences, Faculty of Pure and Applied Sciences, University of The West Indies, Cave Hill Campus, Bridgetown, St Michael, Barbados; 2 Brady School of Medicine, East Carolina University, Greenville, North Carolina, USA; 3 Centre de Recherche de l'Institut Universitaire de Cardiologie et de Pneumologie de Québec, Université Laval, Quebec, Canada

## Abstract

**Background:**

Acylation Stimulating Protein (ASP) has been shown to influence adipose tissue triglyceride (TG) storage. The aim was to examine ethnic differences in ASP and leptin levels in relation to lipid profiles and postprandial changes amongst African American (AA) and Caucasian American (CA) women matched for BMI.

**Methods:**

129 women were recruited in total (age 21 – 73 y): 24 non-obese (BMI < 30 kg/m^2^) CA, 27 obese (BMI ≥ 30 kg/m^2^) CA, 13 obese diabetic CA, 25 non-obese AA, 25 obese AA, and 15 obese diabetic AA. Cholesterol, HDL-C, LDL-C, apoB, glucose and insulin were measured at baseline. TG, non-esterified fatty acids, leptin, and ASP were measured at baseline and postprandially following a fat meal.

**Results:**

ASP, leptin, insulin and TG were significantly increased in obese subjects within each race. However, AA women had significantly lower ASP and TG than CA women at all BMI. Obese and diabetic AA women had significantly lower apoB levels than CA women when compared to their respective counterparts. For AA women, fasting ASP was positively correlated with BMI, cholesterol, apoB, LDL-C and glucose. For CA women, fasting ASP was positively correlated with BMI, leptin, glucose and insulin. However, for any given BMI, ASP was significantly reduced in AA vs CA (p = 0.0004). Similarly, for any given leptin level or TG levels, ASP was significantly lower in AA women (p = 0.041 and p = 0.003, respectively).

**Conclusion:**

CA women have higher baseline TG levels and an earlier TG peak that is accompanied with higher ASP levels suggesting increased ASP resistance, while AA women have lower baseline TG levels and a later TG peak at lower ASP levels suggesting increased ASP sensitivity. This may explain why AA women may have fewer metabolic complications, such as diabetes and CVD, when compared to their Caucasian counterparts at the same level of obesity.

## Background

There has been an increase in the general prevalence of obesity from 15% during NHANES II (1976–1980) to more recent estimates of nearly 31% during NHANES (1999–2000)[1]. There are several risk factors that have been associated with obesity, including hyperinsulinemia, diabetes, hyperlipidemia, hypertension, and cardiovascular disease. In addition to the increased morbidity, approximately 325 000 deaths in the United States each year among nonsmokers are attributed to obesity [2,3]. Approximately 49% of African American women in the US are obese [4]. However, not all overweight women, either African American or Caucasian American, develop the metabolic abnormalities associated with their excess weight. Specifically, while the prevalence of obesity is much higher in African American women than Caucasian American women by approximately 15% [5], and insulin resistance/hyperinsulinemia is also higher [6,7]; the incidence of diabetes is only 5% higher [5]. In addition, prevalence of cardiovascular disease appears to be lower in African American women [8,9]. While it has been suggested that type 2 diabetes is associated with an accelerated rate of development of cardiovascular disease, particularly in African American women [10], nonetheless it is important to note that the mortality rate associated with obesity is actually higher in Caucasian American than in African American women [11]. Therefore, although there is an increased prevalence of risk factors for CVD (obesity and diabetes), this does not result in an equivalent increase in prevalence of CVD in African American women compared to Caucasian women.

Interestingly, when matched for BMI, percent fat, and waist-to-hip ratio or waist circumference, the frequency of cardiovascular disease risk factors associated with abdominal obesity (impaired glucose tolerance and dyslipidemia) is still lower in abdominally obese African American women vs. Caucasian American women [8,9]. Studies suggest that differences in central fat distribution, in particular visceral obesity, may play a role [9]. Nonetheless there still remains a controversy with respect to the differences in the levels and metabolic activity of visceral vs. subcutaneous fat having some, if any, responsibility for the ethnic differences seen between African and Caucasian women [12,13].

In recent years, adipokines have increasingly attracted attention for their potential roles in obesity and related metabolic diseases. Decreased adiponectin is associated with obesity, diabetes, cardiovascular disease and breast cancer [4]. Further, racial differences have been reported in a number of studies, which indicate that AA have lower adiponectin levels that CA [14-16] although not all studies agree [17]. Leptin, the product of the ob gene, is produced by adipose tissue and secreted into the circulation [18]. Leptin functions as a part of a lipostatic signaling pathway that regulates energy homeostasis both centrally and in the periphery (review[19]). Both leptin mRNA and plasma leptin correlate positively with BMI and adiposity in humans [20]. Weight loss in obese subjects results in a decrease in serum leptin concentrations [20]. Circulating leptin concentrations decrease independently of modest changes of body fat content during short-term periods of fasting and are rapidly normalized when the fasting period is terminated [21,22]. To date, comparison of leptin in AA vs CA has produced mixed data, with increased [23,24], decreased [25], or no change in leptin [26-28] between the groups. We have previously demonstrated ethnic differences in both adiponectin and leptin in Asian Indians compared to Caucasians [29]. Differences in inflammatory factors, several of which are also produced by adipose tissue, have also been evaluated. Several recent studies provide varied data with respect to inflammatory factors, one demonstrating that African American women presented with increased C-reactive protein, IL-6 and fibrinogen relative to Caucasian counterparts [30], while another presented data that African American women had lower visceral adipose tissue, insulin sensitivity, TNFalpha and soluble TNFR-1 [31]. Further, studies in adipose tissue have demonstrated that there was no difference in mRNA expression or in adipose tissue explant secretion of IL-8, PGE2 and IL-6 in African American women vs Caucasian women [23].

Acylation stimulating protein (ASP), also an adipokine, is produced within the micro-environment of adipocytes and increases within this environment postprandially as demonstrated using *in vivo *arterial-venous gradients across a subcutaneous adipose tissue bed in humans [32,33]. Furthermore, ASP production and the responsiveness of the adipocyte increased as a result of adipocyte differentiation [34]. ASP acts in an autocrine manner on adipocytes to increase triglyceride synthesis, via stimulation of fatty acid esterification and glucose transport [35]. ASP also inhibits hormone sensitive lipase independently and additively to insulin in adipocytes. These ASP effects are mediated through a G-protein coupled receptor (C5L2) [36,37]. There are several studies that demonstrate a strong correlation between plasma ASP and BMI in a general population [35] although racial differences are unknown.

In the present study we examined ASP and leptin levels in relation to fasting and postprandial lipid profiles amongst non-obese, obese, and obese diabetic African American and Caucasian American women matched for BMI.

## Methods

### Subjects

Six groups of women participated in this study: non-obese (body mass index, BMI < 30) African Americans, non-obese Caucasian Americans, obese (BMI >= 30) African Americans, obese Caucasian Americans, obese diabetic African Americans, and obese diabetic Caucasian Americans. Classification of type 2 diabetes was done with the criteria of the National Diabetes Data Group [38]. Duration of diabetes was greater than three years. Over 40% of those with type 2 diabetes were receiving some form of pharmaceutical therapy in addition to diet and physical activity recommendations, all of which was suspended two weeks prior to sample collection. Data from some of these subjects was used in a previous study that reported only on postprandial TG [39]. The participants were free of vascular disease, cancer or emotional distress, and were not taking any medication that may affect carbohydrate or lipid metabolism. The subjects were not taking hormone replacement therapy or birth control pills. The African Americans group was matched on age and stratified to subjects in the corresponding Caucasian group according to BMI. The women who participated in this study were recruited consecutively over a period of 18 months from the Department of Surgery at East Carolina School of Medicine. African American women were included in this study only if their parents and grandparents were African American descent. Body mass and height were recorded to the nearest 0.1 kg and 0.1 cm, respectively, and BMI calculated. One hundred and twenty-nine women were recruited in total: 24 non-obese Caucasian, 27 obese Caucasian, 13 obese diabetic Caucasian, 25 non-obese African, 25 obese African, and 15 obese diabetic African Americans. Written consent was obtained from all the subjects after they were informed of the nature of the study. The Institutional Review Board for human subject research approved the protocols used in this study.

Fasting blood samples were collected from all the women in the six groups after a 12-hour fast. The plasma was analyzed for triglyceride (TG), total cholesterol (TC), HDL-C, LDL-C, apolipoprotein B (apoB), non-esterified fatty acids (NEFA), glucose, insulin, ASP, and leptin concentrations as described below.

#### Postprandial State

In the second part, we evaluated the postprandial response to a fat-load challenge in 10 non-obese Caucasian, 8 obese Caucasian, 10 non-obese African, and 7 obese African Americans. A blood sample was taken from each volunteer after an overnight fast (basal state). All basal plasma was analyzed for TG, TC, HDL-C, LDL-C, apoB, NEFA, glucose, insulin, ASP, and leptin concentrations. Each volunteer then ingested a fatty meal, as previously described (16). In brief, each subject was given a liquid fatty meal (85.5% of calories from fat), which was prepared from 350 mL heavy whipping cream (39.5% wt/vol fat), 2 tablespoonfuls of chocolate-flavored syrup, 1 tablespoonful of sugar, and 1 tablespoonful of instant nonfat dry milk. Subjects consumed 175 mL of this fatty meal calculated in 1 m2 of body surface area to provide 65 g fat/m2 body surface. The mean dose of the fat meal administered was 197 mL.

Postprandial blood samples were drawn via an indwelling intravenous catheter at 2, 4, 6 and 8 hours after ingestion of the fatty meal. Blood was collected from each subject and a preservative solution containing sodium azide (50 mg/mL) and aprotinin (1 TIU/mL) was added. Plasma was prepared by centrifugation, aliquoted and stored at -80°C until analyzed for TG, NEFA, ASP, and leptin.

### Plasma Analyses

Plasma TG were measured enzymatically on an IL Monarch centrifugal analyzer (Instrumental Laboratory, Warrington, Chesire, UK) with correction for free glycerol [40]. Plasma TC was measured with a commercial enzymatic colorimetric method (cholesterol 50 kit; Sigma, Poole, UK). Plasma NEFA was measured by an enzymatic method (Wako NEFA kit; Alpha Laboratories, Eastleigh, UK). HDL-C concentrations were measured by a commercial enzymatic colorimetric kit (Roche Diagnostics, Laval, Quebec, Canada). Plasma HDL-C was separated according to Gidez et al [41] by heparin/manganese chloride precipitation. Total apoB was measured by a competitive enzyme-linked immunosorbent assay (ELISA), using rabbit anti-human apoB antibody (in house), a commercial standard (837237; Boehringer Mannheim, Laval, Quebec, Canada), and controls (Precipath 1285874 and Precinorm 781827: Boehringer Mannheim). Plasma insulin was measured by microparticle enzyme immunoassay (IMX; Abbott Labs, Abbott Park, IL). Samples were analyzed spectrophotometrically for glucose (16-UV; Sigma Chemical Co., St. Louis, MO). Glucose was assayed in serum samples by a commercial enzymatic colorimetric kit (Sigma, St. Louis, MO). Plasma ASP was assayed by an in-house ELISA assay, using a monoclonal antibody as capture antibody and a polyclonal antibody as detecting antibody as previously described [42,43]. Leptin was measured by radioimmunoassay (Linco, St. Charles, MO).

### Calculations and Statistics

Body mass and height were recorded to the nearest 0.1 kg and 0.1 cm, respectively, and BMI calculated as weight (kg)/height (m)2. Plasma LDL cholesterol was calculated according to the Friedewald equation as evaluated by Schectman, Patsches, and Sasse [44]. To examine the main effects of race and group, a two-way repeated measure analysis of variance was performed (two-way ANOVA, with all pairwise multiple comparisons using Bonferoni t-tests). For the 8 hour fat-load, when the equal variance test failed, data was transformed to the trapezoid area under the curve (AUC), and the means compared by one-way ANOVA. Pearson correlation was performed to examine correlations amongst plasma components. One-way ANOVA was then performed when there was a significant effect of race or group. Statistical analysis was performed with SigmaStat (Jandel, San Rafael, CA). Significance was set at P < 0.05.

## Results

As seen in Table 1, non-obese, obese and obese diabetic AA subjects were matched to CA based on age and body mass index (BMI). BMI for the non-obese subjects was significantly less than the obese and obese diabetic subjects in each race. Fasting plasma hormones and lipid profiles of the subjects are also shown. No significant difference was found for glucose, NEFA, total cholesterol, apoB, LDL-C, or LDL-C/apoB compared within each race between obese and non-obese. TG (p < 0.02), and HDL (p < 0.02) in non-obese subjects were significantly different than their obese and diabetic obese counterparts within each race. Furthermore, glucose, NEFA, total cholesterol, apo B and LDL-C in obese diabetic subjects was significantly different from both their non-obese (p < 0.02) and obese (p < 0.05) counterparts in each race. Interestingly, with CA women, the insulin levels in obese diabetic subjects were significantly different from both their non-obese (p < 0.02) and obese (p < 0.05) counterparts. In AA women, insulin in non-obese subjects was significantly lower than their obese and diabetic obese counterparts. In the diabetic subjects within each race, HDL (p < 0.02) demonstrated a difference only with respect to the non-obese counterparts and not the obese. Further, in AA women, the average TG in the obese diabetic group was significantly different from both the non-obese (p < 0.02) and obese (p < 0.02) matched groups. In the CA women, TG in the obese diabetic group was significantly different only from the non-obese (p < 0.02) group. There was a race effect demonstrated for non-obese and obese groups between AA women and CA women in that AA women had lower TG than CA women. Obese AA women had significantly lower apo B levels than obese CA women. Consequently, AA women had a higher LDL-C/apoB ratio (p < 0.05) than CA women in every group (Table 2), thus showing a race effect.

**Table 1:** Age, BMI, Plasma Hormones and Lipid Profile of Subjects

	**AA women**			**CA women**		
	**Non-obese**	**Obese**	**Obese Diabetic**	**Non-obese**	**Obese**	**Obese Diabetic**

**n**	25	25	15	24	27	13

**Age (yr)**	40.4 ± 2.2	37.0 ± 1.4	45.3 ± 2.0	37.0 ± 2.5	35.2 ± 1.5	39.9 ± 3.1

**BMI (kg/m^2^)**	24.2 ± 0.6	38.6 ± 2.0**	45.9 ± 3.0**	22.5 ± 0.4	46.5 ± 3.1**	47.0 ± 3.3**

**Glucose (mg/dL)**	84.2 ± 2.3	91.2 ± 2.6	209.8 ± 15.5*^a^	85.1 ± 3.3	96.2 ± 5.1	168.5 ± 19.1*^a^

**TG (mg/dL)**	51.7 ± 4.8^††^	78.3 ± .6*^††^	120.0 ± 12.4*^a^	85.5 ± 7.0	123.4 ± 14.1*	131.9 ± 17.1*

**NEFA(mM)**	0.503 ± 0.06	0.643 ± 0.08	1.059 ± 0.162*^a^	0.552 ± 0.05	0.620 ± 0.05	0.799 ± 0.093*^a^

**TC (mg/dL)**	173.7 ± 9.1	158.4 ± 8.3	205.4 ± 9.5*^a^	156.0 ± 5.8	169.5 ± 8.2	193.5 ± 11.7*^a^

**Apo B (mg/dL)**	79.7 ± 8.6	64.7 ± 3.8^†^	97.4 ± 5.6*^a^	75.0 ± 7.2	88.9 ± 9.0	105.0 ± 5.2*^a^

**HDL-C (mg/dL)**	59.6 ± 4.3	40.3 ± 4.1*	37.6 ± 2.8*	50.8 ± 3.6	38.8 ± 3.7*	33.5 ± 2.9*

**LDL-C (mg/dL)**	103.7 ± 8.6^†^	102.5 ± 7.0	143.8 ± 8.2*^a^	88.1 ± 4.1	105.9 ± 7.5	133.6 ± 12.1*^a^

**LDL-C/ApoB**	1.44 ± 0.14^†^	1.63 ± 0.07^†^	1.53 ± 0.05^†^	1.33 ± 0.12	1.31 ± 0.07	1.34 ± 0.08

**Insulin (pM)**	7.8 ± 1.3	17.8 ± 2.4*	19.7 ± 4.6*	6.8 ± 0.8	16.5 ± 1.9*	26.9 ± 4.6*^a^

**Leptin (ng/mL)**	16.4 ± 1.6	54.0 ± 6.9*	45.8 ± 6.7*	20.7 ± 4.8	58.4 ± 5.1*	43.0 ± 4.8*

**ASP(nM) † **	18.6 ± 1.1^††^	26.3 ± 2.4*^††^	27.5 ± 3.1*^††^	26.9 ± 2.5	34.9 ± 2.9*	40.1 ± 4.8*

Values were measured from fasted plasma and are shown as averages ± SEM for each group for CA (Caucasian American) and AA (African American), where * p < 0.05 and ** p < 0.01 for comparison with respect to non-obese subjects or ^a ^p < 0.05 with respect to obese non diabetic subjects within each race. An effect of race was demonstrated where ^† ^p < 0.05 or ^†† ^p < 0.01.

**Table 2:** Pearson Correlations with Respect to ASP and Leptin within CA and AA women

	**AA women**	**CA women**	**AA women**	**CA women**
**R/P**	ASP	ASP	Leptin	Leptin

**n**	65	64	65	64

**BMI**	r = 0.260 p = 0.036	r = 0.357 p = 0.015	r = 0.693 p < 0.0001	r = 0.737 p < 0.0001

**Leptin**	ns	r = 0.362 p = 0.014	-	-

**TG**	r = 0.305 p = 0.014	ns	ns	r = 0.120 p = 0.05

**TC**	r = 0.388 p = 0.002	ns	ns	ns

**Apo B**	r = 0.481 p = 0.001	ns	ns	ns

**HDL-C**	ns	ns	r = -0.301 p = 0.015	ns

**LDL-C**	r = 0.340 p = 0.006	ns	ns	ns

**Glucose**	r = 0.256 p = 0.041	r = 0.248 p = 0.047	ns	ns

**Insulin**	ns	r = 0.358 p = 0.004	r = 0.219 p = 0.04	ns

Values of correlation coefficient (R) and p values (P) are shown for significant correlations only, where ns indicates non-significant.

**Figure 1:**
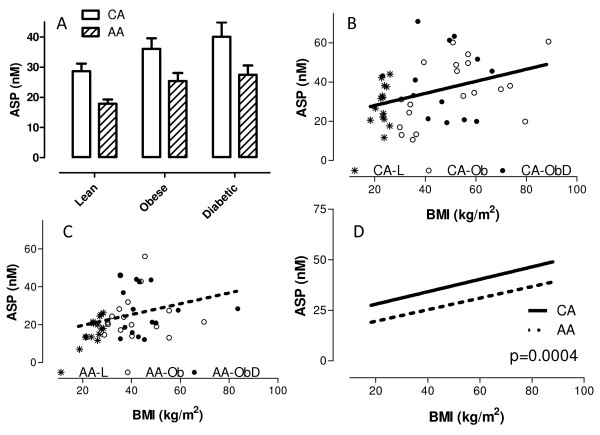
**Fasting ASP values in non-obese, obese and obese diabetic Caucasian American (CA) and African American (AA) women relative to BMI**. Fasting blood samples were collected and ASP measured in CA (n = 64) and AA (n = 65) women. Panel A: Average ± SEM is given for each group where * p < 0.05 vs. respective lean control group, and ^†† ^p < 0.01 for AA vs. CA. Panels B and C: Correlation between plasma ASP and BMI is given for CA (Panel B, p = 0.015), AA (panel C, p = 0.036). Panel D: Correlation between ASP and BMI was significantly different between AA and CA (p = 0.0004).

With respect to hormones, the average levels of insulin (p < 0.02) and leptin in non-obese subjects were significantly lower than their obese counterparts within each race. Obese and obese diabetic individuals had significantly higher levels of ASP (p < 0.02) than their non-obese counterparts for each race (Figure 1A). In all groups (non-obese, obese and obese diabetic) AA women had significantly lower ASP levels (p < 0.006) than CA women. A race effect was not apparent for the other parameters measured, including leptin. ASP was the only hormone-like parameter that consistently demonstrated a race effect.

Table 2 presents Pearson correlation analysis between ASP and various plasma parameters. In AA women, ASP strongly correlated with lipid parameters (TG, cholesterol, apoB and LDL-C), while in CA women there were only correlations between ASP and leptin or insulin. A significant positive correlation between ASP and BMI was present in both races (CA p = 0.02, AA p = 0.036, Figure 1B and 1C). Interestingly, for any given BMI (ranging from non-obese to obese), ASP is always less in AA vs. CA women (Figure 1D, p < 0.0004). Similarly, for any given plasma TG level, ASP is always lower in AA (p < 0.003), and for any given leptin level, ASP is always lower in AA (p = 0.041).

Pearson correlation analysis between leptin and plasma parameters was also performed (Table 2). Leptin positively correlated with BMI in both races (AA p < 0.0001, CA p < 0.0001). Interestingly, there was a difference between AA and CA women in that leptin was significantly correlated with HDL-C (r = -0.301, p = 0.015) and insulin (r = 0.219, p = 0.04) only in AA women. In CA women, leptin was marginally correlated to TG.

**Figure 2:**
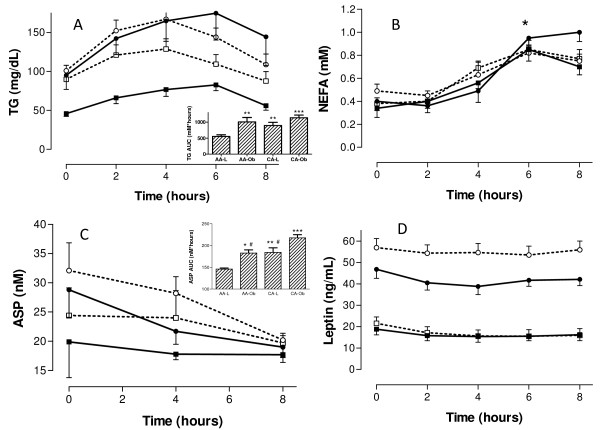
**Postprandial changes in plasma triglycerides, non-esterified fatty acids, ASP and leptin**. Blood samples were collected after a 12 hour fast and then subsequently at 2, 4, 6, and 8 hours after ingestion of a fatty meal in 4 groups: African American non-obese women (AA-L, filled squares, solid line, n = 10). African American obese women (AA-O, filled circles, solid line, n = 7), Caucasian American non-obese women (CA-L, open squares, dotted line, n = 10), and Caucasian American obese women (CA-O, open circles, dotted line, n = 8) for triglyceride (A), non-esterified fatty acids (B), ASP (C) and leptin (D) expressed as averages ± SEM. Non-obese and obese groups were matched for BMI between races. Significant differences were as follows: panel A: TG time course p < 0.01 for AAL vs AAO and CAO, and TG area-under-the curve (AUC)(insert) ** P < 0.01 *** p < 0.001; Panel B: NEFA time course: * p < 0.01 for 6 hours vs. zero, 2 hours and four hours; Panel C: ASP time course and ASP AUC (insert). There was a significant race affect with CA-L and CA-O women having significantly greater ASP AUC than the AA counterparts, * p < 0.05, ** p < 0.01 and *** 0.001 vs AA-L and # p < 0.05 vs. CA-O; Panel D: Leptin time course was significantly differences between non-obese and obese subjects within each race (AA: p < 0.001, CA: p < 0.001), further, AA-O were significantly lower than CA-O at all time points, p < 0.001.

A fat load was given to four groups of subjects (non-obese AA: n = 7, obese AA: n = 10, non-obese CA: n = 8, obese CA: n = 10). The AA and CA groups were matched for BMI. TG, NEFA, leptin and ASP were measured at fasting and up to eight hours after the meal. As shown in Figure 2A, all groups demonstrated a similar pattern of NEFA. The levels of NEFA peaked at six hours (p < 0.01 compared to zero, 2 hours and 4 hours).

By contrast, there were significant differences in the TG curves, as seen in Figure 2B. In general, non-obese AA women had lower plasma TG levels than non-obese CA women at basal and 2, 4, 6, and 8 hours postprandially. Obese subjects had a higher area-under-the-curve for their TG levels after the fatload curve when compared to their respective non-obese counterparts (p = 0.0008) for both AA and CA women (AA non-obese vs AA obese p < 0.01 and CA non-obese vs. CA obese p < 0.01 by RM-ANOVA). Plasma TG in AA women (non-obese and obese) peaked at six hours (AA: p < 0.05 for 6 hours vs. 8 hours), while in CA women it peaked at four hours (p < 0.01 for 4 hours vs. 8 hours). There was a significant difference observed between races (AA vs CA: p = 0.006) and body size (obese vs non-obese: p < 0.0001) related to the total area-under-the-curve for postprandial TG over time. Baseline levels of TG were correlated with the total area under the TG time curve for all women (r2 = 0.531, p = 0.001).

ASP and leptin were followed during the course of the fat load. The levels of leptin (Figure 2D) did not change significantly over time. Not surprisingly, there were differences between non-obese and obese subjects within each race (AA: p < 0.001, CA: p < 0.001). Although there was no difference in the leptin time curves between non-obese AA women and non-obese CA women, obese AA women had significantly lower levels of leptin at all time points when compared to obese CA women (p < 0.001).

AA women and CA women demonstrated different patterns for ASP time curves (Figure 2C). Both non-obese and obese AA women tended to have lower basal ASP levels and a more rapid decline in ASP from basal to four hours as well as from basal to eight hours when compared to the CA women (p = 0.047). Fasting levels of ASP positively correlated with the decrease in ASP levels over 8 hours for both AA and CA women (r = 0.898, p < 0.001 for AA and r = 0.969, p < 0.001 for CA), with the higher the fasting ASP, the greater the decrease at 8 hours. The drop in ASP from baseline to 8 hours correlated with basal TG (r = 0.620, p = 0.006) in CA women only. In addition, the area under the ASP time curve was significantly greater in obese women when compared to their non-obese counterparts within race (AA non-obese vs. AA obese, p < 0.05, CA non-obese vs. CA obese p < 0.05). Furthermore, AA women demonstrated lower postprandial ASP AUC than CA women regardless of their BMI (Figure 2C inset, non-obese AA vs. non-obese CA, p < 0.01 and obese AA vs. obese CA, p < 0.001 by ANOVA).

## Discussion

Although the prevalence of obesity is greater in African American women than in Caucasian American women, AA women do not necessarily have a corresponding proportional increase in the risk of metabolic complications associated with obesity when both groups are matched for BMI, body fat, waist circumference or waist-to-hip circumference ratio [9]. In the present study, within each race, the average levels of insulin, leptin, ASP, TG, and HDL-C in non-obese subjects were significantly different from their obese counterparts. Notably, a race difference was evident whereby the AA women had significantly lower ASP, fasting TG, and apoB levels than CA women at all BMI. Furthermore, TG time curve profiles showed similar trends within race but differed between races. The area-under-the-curve for TG was lower in non-obese AA women indicating faster TG clearance. In addition, ASP AUC was positively correlated with baseline ASP levels, with lower ASP AUC in AA women. Moreover, in AA women, ASP positively correlated with BMI, TG, TC, apoB, LDL-C and glucose, whereas for CA women, ASP positively correlated with BMI, leptin, glucose and insulin. Taken together, this study suggests that AA women have more efficient TG clearance with lower ASP levels, suggesting increased ASP sensitivity.

The question remains as to what biochemical factors may explain the difference between AA women and CA women noted above. Adipose tissue stores are determined by the balance between fat utilization and fat storage. AA women have been reported to show decreased *in vivo *fatty acid oxidation [45], a state that was shown *in vitro *to be associated with lower skeletal muscle FA oxidation [46]. Non-obese AA have decreased levels of circulating adiponectin as well, an adipokine associated with increased fatty acid oxidation [16]. These factors may enhance shunting of fuel from muscle to adipose tissue for storage, and contribute to the development and maintenance of obesity in AA women.

In adipose tissue, the balance of TG stores is determined by the opposing reactions of TG synthesis and lipolysis. While a lower basal lipolytic rate in adipose tissue from AA women vs CA women has been demonstrated [47,48] stimulation of hormone-stimulated lipase resulted in equal lipolytic activity between races [39]. Neither differences in beta-adrenergic receptors nor eNOS (both of which can influence lipolysis) could explain the AA vs CA difference [49,50], although the potential exists for a greater inhibition of lipolysis by alpha-adrenerigc receptors in obese AA women[51]. Overall, this suggests that changes in lipolysis may not explain the racial differences in adipose tissue maintenance.

The differences in the metabolic abnormalities between AA women and CA women may be related to racial differences in the rate of triglyceride clearance and adipose tissue storage. Both non-obese and obese AA women have an increased capacity to synthesize TG in omental adipose tissue compared to Caucasian American women [13]. In a previous study by Barakat and colleagues, in a separate group of women, lipoprotein lipase mass was shown to be increased in subcutaneous adipose tissue from obese AA women, and postheparin plasma LPL activity was higher in non-obese AA than in non-obese CA, and was associated with a more rapid clearance of postprandial triglycerides [39]. Further, increased mRNA and protein of CD36 (a fatty acid transporter), FATP4 (fatty acid binding protein) and PPARγ in AA vs CA women in visceral fat may also contribute to increase capacity for fatty acid uptake and increased TG storage [52].

ASP has previously been shown to be positively correlated to BMI, with obese individuals having higher ASP levels [35]. This was also true in both AA and CA women. ASP directly stimulates TG synthesis through enhanced fatty acid esterification and glucose transport in human adipocytes and preadipocytes [35]. ASP also increases the efficiency of LPL action by enhancing uptake of fatty acids and protecting LPL from product inhibition [53]. ASP has been shown to increase postprandially within the micro-environment of the adipose tissue, correlating with increased TG uptake into adipose tissue [32,43]. In a recent study, fasting ASP was shown to be directly correlated with postprandial TG clearance in both men and women [54,55] with a lower fasting ASP associated with a more efficient TG clearance. Further, changes in metabolic function, such as thyroid status where hypothyroidism is associated with increased BMI and ASP [56], or in rosiglitazone treatment, which improves metabolic function in type 2 diabetic subjects [57], also resulted in a decreased fasting and postprandial adipose tissue production of ASP. As the function of ASP is to increase adipose tissue TG esterification, and as AA circulating ASP levels remain lower, this could suggest that AA also have increased ASP sensitivity.

## Conclusion

In conclusion, the differences in ASP and TG profiles between races supports that there are race differences in lipid metabolism. The results suggest that AA women have a more efficient synthetic capacity as documented previously by increased triglyceride synthesis, LPL, CD36, FATP4, and PPARγ and, in the present stufy, lower circulating ASP, suggesting increased ASP sensitivity. Overall, AA women may have a more efficient fatty acid trapping than CA women and this may contribute both to development and maintenance of obesity, and to the lower proportional increase in metabolic complications such as diabetes and CVD in African American women compared to their Caucasian counterparts at the same level of obesity.

## Abbreviations

ASP: acylation stimulating protein; AA: African American; CA: Caucasian American.

## Competing interests

The authors declare that they have no competing interests.

## Authors' contributions

TS and JB were responsible for collection of samples, laboratory assays and data analysis. KC and HB were responsible for supervision. All authors contributed to the interpretation, and manuscript preparation and all authors have agreed to the final manuscript.
